# Sparse Representation for Tumor Classification Based on Feature Extraction Using Latent Low-Rank Representation

**DOI:** 10.1155/2014/420856

**Published:** 2014-02-11

**Authors:** Bin Gan, Chun-Hou Zheng, Jun Zhang, Hong-Qiang Wang

**Affiliations:** ^1^College of Information and Communication Technology, Qufu Normal University, Rizhao 276800, China; ^2^College of Electrical Engineering and Automation, Anhui University, Hefei 230000, China; ^3^Intelligent Computing Lab, Institute of Intelligent Machines, Chinese Academy of Sciences, Hefei 230000, China

## Abstract

Accurate tumor classification is crucial to the proper treatment of cancer. To now, sparse representation (SR) has shown its great performance for tumor classification. This paper conceives a new SR-based method for tumor classification by using gene expression data. In the proposed method, we firstly use latent low-rank representation for extracting salient features and removing noise from the original samples data. Then we use sparse representation classifier (SRC) to build tumor classification model. The experimental results on several real-world data sets show that our method is more efficient and more effective than the previous classification methods including SVM, SRC, and LASSO.

## 1. Introduction

Tumor is a solid lesion caused by the abnormal growth of cells. A timely accurate treatment is very important clinically. The premise of an accurate treatment is an exact diagnosis due to the heterogeneity of cancer. That is, we need to classify them accurately before treating tumors. Current methods for classifying cancer malignancies mostly rely on a variety of morphological, clinical, or molecular variables. Despite recent progresses, there are still many uncertainties in diagnosis. The advent of DNA microarray and RNA_seq [1] makes it possible to analyze tumor samples and classify them based on gene expression profiles. Moreover, we can get the expression data of tens of thousands of genes through DNA microarray or RNA-seq simultaneously.

Many methods for molecular data classification or clustering based on gene expression data have appeared in this area [2–14]. Huang and Zheng used independent component analysis [5] to extract features; Gao and Church introduced sparse nonnegative matrix factorization for feature extraction [4]; Zheng et al. proposed metasample-based sparse representation [7], and Furey et al. used support vector machines [8] to classify the gene expression data. All these methods have achieved impressive classification performances.

Recently published sparse representation classification (SRC) is also a powerful tool for processing gene expression data. SRC method was inspired by many theories such as Basis pursuing [15], compressive sensing for signal reconstruction [16], and least absolute shrinkage. It has already been widely used in face recognition [17] and texture classification [18]. In SRC method, test samples can be only represented as a sparse linear combination of the training samples from the same class. Furthermore, an imposed *l*_1_-regularized least square optimization is used to calculate an SR coefficient vector with only a few significant coefficients. In theory, a test sample can be well represented by only using the training samples from the same class. However, there is too much noise in gene expression data, which causes that the discriminative features are not obvious and the test samples can also be represented by some training samples from different classes. This will decrease the classification accuracy. To reduce noise [19–21] and get salient features [20] for tumor classification, in this paper, we introduce latent low-rank representation to preprocess gene expression data. By combining it with SRC algorithm, we propose a new method for tumor classification.

Latent low-rank representation (LatLRR) is a kind of theory which can be used to extract principal and salient features from original data. LatLRR is the improved version of LRR. The two methods can be resolved by the inexact augmented Lagrange multiplier (ALM) optimization. In [19–22], LRR has been successfully used for the recovery of subspace structure, subspace segmentation, feature extraction, outlier detection, and so forth. In [23], the author introduced LRR theory for face recognition in order to remove noise and achieved an impressive result. Based on these successful applications, in this paper, we introduce LatLRR into sparse representation classifier for tumor classification. Firstly, we use LatLRR to remove noise from original data and extract salient features. Then based on the new extracted salient features, we design sparse representation classifier to classify new test samples. We referred to the proposed method as SRC-based latent low-rank representation (SRC- LatLRR).

The rest of the paper is organized as follows.  Section 2 describes our proposed SRC-LatLRR method in detail. We firstly review SRC and latent low-rank representation methods in Sections 2.1 and 2.2, respectively. Then we present our method in detail in Section 2.3.  Section 2.4 specifies our experimental setting. In Section 3, we evaluate our method using several publicly available gene expression data sets.  Section 4 concludes the paper and outlines our future work.

The abbreviations used in this paper are summarized in the Abbreviations section.

## 2. Methods

### 2.1. Sparse Representation Classification

Sparse representation classification is a supervised classification. Let *W* ∈ *R* ^*m*×*n*^ denote a training sample matrix with *n* samples and *m* genes. As we know, each DNA microarray chip usually contains thousands of genes; the number of genes is much larger than tumor samples; that is, *m* ≫ *n*.

Let *c*_*l*_ be the *l*th sample of *W* and the *n* samples are divided into *k* object classes. Assuming that there are *n*_*i*_ samples belonging to *i*th class and making up *W*_*i*_ = [*c*_*i*,1_, *c*_*i*,2_,…, *c*_*i*,*n*_*i*__], the whole data set can be reexpressed as *W* = [*W*_1_, *W*_2_,…, *W*_*k*_]. Suppose that a new testing sample *y* ∈ *R* ^*m*^ belongs to *i*th class. Based on the theory of sparse representation, *y* would lie in the linear span of the training samples *W*_*i*_; that is,

(1) $$\begin{array}{c} y=\alpha_{i,1}c_{i,1}+\alpha_{i,2}c_{i,2}+\cdots +\alpha_{i,n_{i}}c_{i,n_{i}}, \end{array}$$

where *α*_*i*,*j*_ ∈ *R* is a scalar and *j* = 1,2,…, *n*_*i*_.

Supposing a linear representation coefficient vector *x*_0_ ∈ *R* ^*n*^,  *y* can be also rewritten as

(2) $$\begin{array}{c} y=Wx_{0}. \end{array}$$

Ideally, if the training samples are sufficient and the training samples sets that belong to different class are disjoint each other, then we have

(3) $$\begin{array}{c} x_{0}=\left[0,\ldots ,0,\alpha_{i,1},\alpha_{i,2},\ldots ,\alpha_{i,n_{i}},0,\ldots ,0\right]\in R^{n}; \end{array}$$

that is, in *x*_0_, only the entries corresponding to the same class as *y* are nonzero.

From the above analysis, it can be seen that we can classify the test sample *y* according to *x*_0_. So the key problem is how to calculate *x*_0_ in (2). As in [7], *x*_0_ would be sparse if the number of object classes *k* is large; this is what sparse representation implies. According to the theory of compressive sensing [16, 24–26] and SR, *x*_0_ can be achieved by solving the following *l*_1_-minimization problem:

(4) $$\begin{array}{c} {\overset{\wedge }{x}}_{1}=\arg\;\min{||x||}_{1}\;\text{s}.\text{t}.\;\;Wx=y. \end{array}$$

This problem can be solved by standard linear programming methods [15]. But (4) has no exact solutions since *m* ≫ *n*. Then a generalized version of (4) can be conceived:

(5) $$\begin{array}{c} J\left(x,\lambda \right)=\underset{x}{\min}\left\{{||Wx-y||}_{2}+\lambda {||x||}_{1}\right\}, \end{array}$$

where *λ* is a scalar regularization. This function can balance the degree of noise by using *λ*. In this study, we solve this function by the truncated Newton interior-point method [27].

### 2.2. Latent Low-Rank Representation

Latent low-rank representation is an extension of low-rank representation. Consider an observed data matrix *X* = [*x*_1_, *x*_2_,…, *x*_*n*_] ∈ *R* ^*D*×*n*^, where each column vector *x*_*i*_ is a sample, and a dictionary *A* = [*a*_1_, *a*_2_,…, *a*_*m*_] ∈ *R* ^*D*×*m*^, where *a*_*i*_ is also a sample. *X* can be linearly represented by the dictionary. That is,

(6) $$\begin{array}{c} X=AZ, \end{array}$$

where *Z* = [*z*_1_, *z*_2_,…, *z*_*n*_] ∈ *R* ^*m*×*n*^ is a coefficient matrix and each *z*_*i*_ is the representation of *x*_*i*_. Equation (6) means that each column vector of *X* can be represented by a linear combination of the bases in *A*. In (6), the dictionary *A* should be overcomplete enough to represent any observed data matrix *X*. But meanwhile, this causes multiple feasible solutions of *Z* to (6). To achieve the optimal solution, low rankness criterion is introduced to (6):

(7) $$\begin{array}{c} \underset{Z}{\min}\mathrm{rank}\left(Z\right),\;\text{s}.\text{t}.\;\;X=AZ. \end{array}$$

Here, the optimal solution Z* is the so-called lowest-rank representation of data *X* with respect to the dictionary *A*. Unfortunately, function (7) can not be easy to solve because of the discrete nature of the rank function. By matrix completion method [28–30], we replace solving low-rank problem with dealing with nuclear norm [31]; then problem (7) can be rerepresented as

(8) $$\begin{array}{c} \underset{Z}{\min}\;{||Z||}_{*},\;\text{s}.\text{t}.\;X=AZ, \end{array}$$

where ||*Z*||_∗_ means the nuclear norm of matrix *Z*, that is, the sum of the singular values of matrix *Z*.

Strictly speaking, the dictionary *A* should be overcomplete and noiseless. But this kind of dictionary is difficult to get. In practice, we usually use observed data matrix *X* itself as the dictionary [19, 21, 32]. Finally we have the following convex optimization problem:

(9) $$\begin{array}{c} \underset{Z}{\min}\;{||Z||}_{*},\;\text{s}.\text{t}.\;X=XZ. \end{array}$$

To solve this equation, two conditions need to be met. Firstly, the data sampling *X* should be sufficient. Secondly, the data sampling *X* should also contain sufficient noiseless data to achieve robust capability. In fact, the first one can be easily met but the second one not. Because gene expression data are usually noisy, in reality, function (9) may be invalid and not robust.

To solve the problem in (9), we introduce the following LRR problem [20]:

(10) $$\begin{array}{c} \underset{Z}{\min}\;{||Z||}_{*},\;\text{s}.\text{t}.\;X_{O}=\left[X_{O},X_{H}\right]Z, \end{array}$$

where *X*_*O*_ is the observed data matrix and the *X*_*H*_ is the unobserved data, that is, the hidden data. We use the concatenation of *X*_*O*_ and *X*_*H*_ as a dictionary. The optimal result of (10) is *Z*_*O*,H_* = [*Z*_*O*∣H_*; *Z*_*H*∣O_*], where *Z*_*O*∣H_* and *Z*_*H*∣O_* correspond to *X*_*O*_ and *X*_*H*_, respectively.

By solving (10), the two problems above can be solved well. Then our next mission is to recover the affinity matrix *Z*_*O*∣H_* by using only *X*_*O*_ in the absence of the hidden data *X*_*H*_. The method is called latent low-rank representation (LatLRR), which is an improvement of LRR.

Supposing we have two matrices *X*_*O*_ and *X*_*H*_, then by solving (10) we have the following equations:

(11) $$\begin{array}{c} Z_{O\;|\;H}^{*}=V_{O}V_{O}^{T},\;\;Z_{H\;|\;O}^{*}=V_{H}V_{O}^{T}, \end{array}$$

where *V*_*H*_ and *V*_*O*_ can be obtained through computing the skinny singular value decomposition of [*X*_*O*_, *X*_*H*_] = *U*∑*V* ^*T*^, and *V* = [*V*_*O*_; *V*_*H*_]. Namely, *X*_*O*_ = *U*∑*V*_*O*_^*T*^ and *X*_*H*_ = *U*∑*V*_*H*_^*T*^.

Depending on function (11), we have

(12) XO=[XO,XH]ZO,H∗=XOZO ∣ H∗+XHZH ∣ O∗=XOZO ∣ H∗+XHVHVOT=XOZO ∣ H∗+U∑VHTVHVOT=XOZO ∣ H∗+U∑VHTVH∑−1UTXO.

Let *L*_*H*|O_* = *U*∑*V*_*H*_^*T*^ *V*_*H*_∑^−1^ *U* ^*T*^; then we have the following simple function:

(13) $$\begin{array}{c} X_{O}=X_{O}Z_{O\;|\;H}^{*}+L_{H\;|\;O}^{*}X_{O}. \end{array}$$

If *X*_*O*_ and *X*_*H*_ come from the same collection of low-rank subspaces, then both *Z*_*O*∣H_* and *L*_*H*∣O_* should be of low-rank, so we can achieve

(14) min⁡ZO ∣ H,LH ∣ O rank⁡(ZO ∣ H)+rank⁡(LH ∣ O)s.t. XO=XOZO ∣ H+LH ∣ OXO.

Just as in [28–30], we also change the above rank minimization problem to the nuclear norm. Then we have the following convex optimization problem:

(15) $$\begin{array}{c} \underset{Z,L}{\min}\;{||Z||}_{*}+{||L||}_{*}\;\text{s}.\text{t}.\;\;X=XZ+LX. \end{array}$$

Here, we replace *X*_*O*_, *Z*_*O*∣*H*_, and *L*_*H*∣*O*_ with *X*, *Z*, and *L*, respectively, for ease of representation. In (15), *X* is the noiseless observed data. By considering there may exist corrupted data or noise in *X*, we also need to introduce a denoising model about (15); then we have

(16) $$\begin{array}{c} \underset{Z,L}{\min}\;{||Z||}_{*}+{||L||}_{*}+\lambda {||E||}_{1}\;\text{s}.\text{t}.\;\;X=XZ+LX+E, \end{array}$$

where *λ* > 0 is a scalar and ||*E*||_1_ is the *l*_1_-norm of sparse noise matrix *E*. If *λ* → +*∞*, the problem (16) will be equivalent to (15), that is, no noise in the observed data *X*. In (16), the optimal solutions XZ*, L*X, and E*  represent the principal features, salient features, and noise, respectively.

To solve the LatLRR problem listed in (16), we introduce the augmented Lagrange multiplier (ALM) [33] method and revise (16) as follows to meet the requirement of ALM algorithm:

(17) min⁡Z,L,J,S,E ||Z||∗+||L||∗+λ||E||1 s.t.  X=XZ+LX+E,               Z=J,  L=S.

This problem can be solved by ALM method which minimizes the following augmented Lagrange function:

(18) ||J||∗+||S||∗+λ||E||1+tr⁡(Y1T(X−XZ−LX−E)) +tr⁡(Y2T(Z−J))+tr⁡(Y3T(L−S)) +μ2(||X−XZ−LX−E||F2+||Z−J||F2+||L−S||F2),

where tr⁡(·) and ||·||_*F*_ denote the trace and Frobenius norm of a matrix, respectively. *μ* > 0 is a penalty parameter. More details about (18) can be found in [33].

### 2.3. Sparse Representation Classification Based on LatLRR

Since LatLRR can extract the salient features and remove noise from original data sets, in this study, before using observed data for classification, we firstly use LatLRR to suppress noise and get the salient features. Then we use the denoised data for tumor classification; that is, we factorize the observed data *X* into

(19) $$\begin{array}{c} X=XZ+LX+E. \end{array}$$

Here, we only use *D* = *LX* for data classification. For a test sample *y*, we can calculate its SR by the following function:

(20) $$\begin{array}{c} J\left(x,\lambda \right)=\underset{x}{\min}\left\{{||Dx-Ly||}_{2}+\lambda {||x||}_{1}\right\}, \end{array}$$

where the parameter *λ* > 0 can be determined experimentally and *x* is a coefficient vector. Assuming the test sample *y* belongs to one of target classes, the training data set is sufficient. When classifying *y*, we introduce *Ly*, where *L* is a square matrix obtained through LatLRR method when extracting the salient features.

Ideally, *Ly* can be linearly represented by the samples from the same class in *D*. Namely, the representation vector *x* should be sparse and the nonzero entries are associated with the columns of *D* from the same class. This will lead us to classify the test samples. However, noise and modeling errors will also introduce some nonzero entries to *x* which correspond to the columns of *D* from the multiple classes [17]. To solve this problem, we classify *Ly* based on how well it can be reconstructed by using the coefficients from each class as in [17].

Using the result of (20), we construct *δ*_*i*_(*x*) as the characteristic function which selects the coefficients associated with the *i*th class in the coefficient vector *x*. By only using *i*th class coefficients to reconstruct the test sample *Ly* as ${\hat{y}}_{i}=D\delta_{i}(x)$, we can classify *Ly* into the minimum residual class between *Ly* and $\hat{y}$; that is,

(21) $$\begin{array}{c} \underset{i}{\min}\;r_{i}\left(y\right)={||Ly-D\delta_{i}\left(x\right)||}_{2}. \end{array}$$

Our classification algorithm can be summarized as follows.

 
*Input.* Observed data *X* ∈ *R* ^*m*×*n*^ for *k* classes; test sample *y*.
   
*Step* *1.* Normalize the columns of *X*.
   
*Step* *2.* Extract the salient features of *X* and remove to some extent noise to get data *D* defined in (19).
   
*Step* *3.* Solve the optimization problem defined in (20).
   
*Step* *4.* Compute the residuals *r*_*i*_(*y*) = ||*Ly*−*Dδ*_*i*_(*x*)||_2_.
 
*Output*. Identity(*y*) = arg min_*i*_ *r*_*i*_(*y*).

Our method can be seen as the combination of SRC [17] and latent low-rank representation for feature extraction [20], so we named it as SRC-LatLRR. In SRC, the test sample is represented as a sparse linear combination of the training samples from the same class. In LatLRR, noise is removed to some extent and salient features are simultaneously extracted from the training samples. So the introduction of LatLRR can improve the classification accuracy of SRC in a way.

### 2.4. Evaluation of the Performance

To evaluate our proposed method, we compare our method with SRC [17, 34], LASSO [35], and SVM [8, 36, 37]. SVM has been proved to be one of the best classifiers for classifying data in the area of “high dimensionality and small sample size” [36, 37]. We do binary classification and multiclass classification experiments in Sections 3.1 and 3.2, respectively. During the experiment, the best results of SRC, LASSO, and SVM are also used to compare with those of our method, which were achieved by choosing appropriate parameters experimentally. As the number of tumor sample is too small, we use stratified 10-fold cross validation in all our experiments. In the multiclass classification experiments, we do not use LASSO method because it is designed only for binary class classification problems [35]. As we know, dimensionality reduction can improve the classification performance and computing speed, so we reduce data dimensionality using between-category to within-category sums of squares methods in our experiments.

## 3. Experimental Results

### 3.1. Two-Class Classification Problem

In this subsection, three two-class microarray data sets are used to evaluate our method: colon cancer [38], prostate cancer [39], and diffuse large B-cell lymphoma [40].

The colon data set contains 62 samples consisting of 40 tumor and 22 normal. The prostate data set contains prostate tumors and normal prostate samples, each consisting of the expression levels of 12600 genes. For the DLBCL data set, the gene expression values were measured by high-density oligonucleotide microarrays. An overview of the three data sets is given in Table 1.

The classification results by using SVM, LASSO, SRC, and the proposed SRC-LatLRR are listed in Table 2. From Table 2, we can see that our method SRC-LatLRR performs well on all the three data sets. Even the performance of SRC-LatLRR is not better than SRC on the prostate cancer data set, but it is better than SVM and LASSO. In summary, SRC has an advantage for the prostate cancer and DLBCL data sets, but SRC-LatLRR is the best classifier for the colon cancer and DLBCL data sets.

To further evaluate our method, in this experiment, we also introduced BW feature selection in our method to classify these three data sets. The results are listed in Table 3, and the number of genes selected is given in the parenthesis behind data set. From Table 3, we can see that after feature selection, our proposed classification method outperforms the other three classification methods, and it can even achieve an accuracy of 100% for the DLBCL data set.

### 3.2. Multiclass Classification Problem

In this subsection, we use four multiclass data sets to further check the classification performance of SRC-LatLRR. The four data sets are lung cancer [41], leukemia [42], 11_tumors [43], and 9_tumors [44].

In lung cancer data set, there are four classes of lung cancer and normal class. This data set contains 203 samples. For leukemia data set, all the samples are classified into acute myelogenous leukemia, acute lymphoblastic leukemia, or mixed-lineage leukemia. The data set includes 72 samples with 11225 genes. For 11_tumors, there are 11 classes of samples, which are ovary, bladder/ureter, breast, colorectal, gastroesophagus, kidney, liver, prostate, pancreas, adeno lung, and squamous lung. This data set includes 174 samples. For the 9_tumors data set, there are 60 samples with 5726 genes. These 9 types of tumors are non-small-cell lung, colon, breast, ovarian, leukemia, renal, melanoma, prostate, and central nervous system. The detailed descriptions about these four data sets are listed in Table 4. All the four data sets were produced by oligonucleotide microarrays and the analysis tool Affymetrix GENECHIP [36].

The experimental results are listed in Table 5. From these results, we can see that the proposed method SRC-LatLRR does not have a clear advantage over SVM and SRC. The reason may be that in these data sets, the training samples of each class are very few so that the sample space is not complete.

We then introduced BW feature selection before applying our method. The obtained results are listed in Table 6. From the results we can see that the proposed method classified leukemia well. For the other data sets, it has no clear advantage. But it performed better than SRC for all the four data sets.

### 3.3. The Choice of the Balanced Parameter

**Figure 1:**
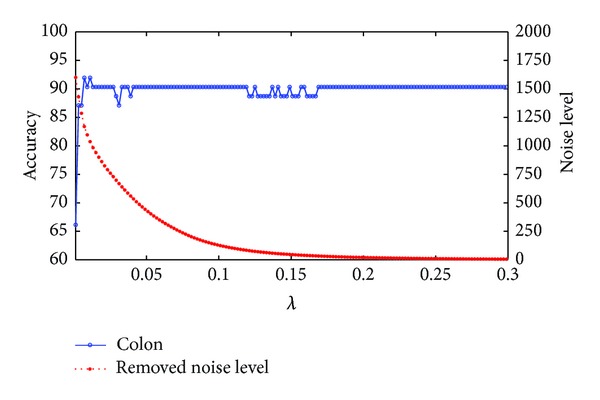
The changing curves of classification accuracy and removed noise level with *λ* on the colon data set.

**Figure 2:**
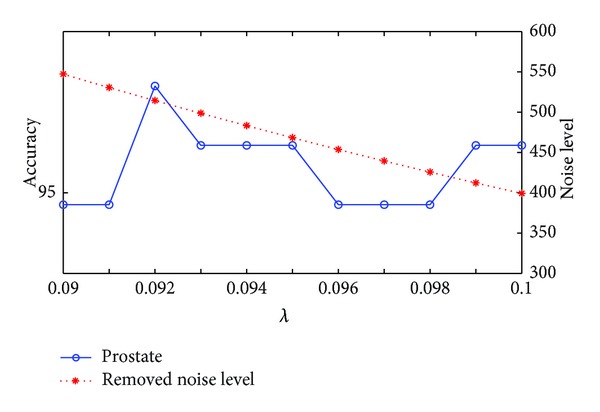
The changing curves of classification accuracy and removed noise level with *λ* on the prostate data set.

**Figure 3:**
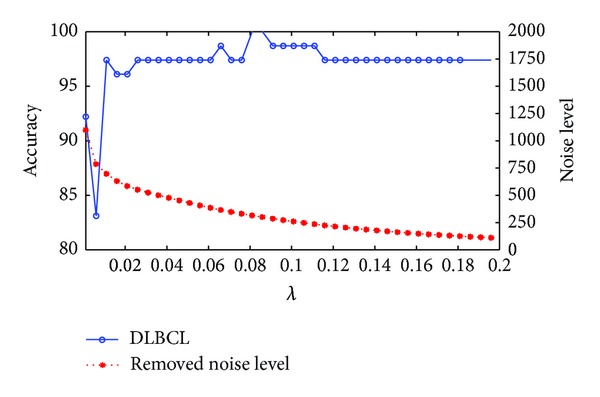
The changing curves of classification accuracy and removed noise level with *λ* on the DLBCL data set.

In this section, we use the data sets described in Section 3.1 to check how *λ* in (16) affect the classification performance. We show the accuracies and the removed noise level by our method at different values of *λ* in Figures 1, 2, and 3 for the colon, prostate, and DLBCL data sets, respectively. From (16), we know that the lower the *λ* is, the bigger the noise level is removed. For these three figures we use ||*E*||_1_ to represent the level of the removed noise. From these three figures we can see that the noise that we remove from the original data can not be too much, or it will reduce the accuracy. The reason is that if *λ* is set to be too small, useful information may be also removed besides noise. On the contrary, if *λ* is too big, the noise that was removed is too little, and we still can not get a good classification result. The experiment suggests that for colon data sets, *λ* = 0.011 is the best choice and *λ* = 0.096 and *λ* = 0.1 for the prostate and DLBCL data sets, respectively.

## 4. Conclusions

For gene expression data, cancer diagnosis is one of the most important clinical applications. In this paper, we have proposed a new SR-based method for tumor classification which uses the noiseless salient features extracted from the original samples to classify a test sample. We compared our method with several state-of-the-art methods including SVM, LASSO, and SRC on seven data sets. The results of experiments show that the proposed method is better than SVM, LASSO, and SRC in a way. These demonstrate that SRC-LatLRR is effective and efficient for tumor classification. We also introduced gene selection into our method. The results show that gene selection can improve the classification accuracy to some extent.

During the study we also found that, for the optimal result of LatLRR on the observed samples, Z* represents the affinity matrix of samples [21]. In theory, the affinity matrix can be used to cluster samples. In future, we will extend it to investigate the property of sample clusters.

## Figures and Tables

**Table 1:** Three binary data sets used in the experiments.

Datasets	Samples		Genes
	Class 1	Class 2	
Colon cancer	40	22	2000
Prostate cancer	77	59	12600
DLBCL	58	19	5469

**Table 2:** Classification accuracies by different methods for the three binary data sets.

Datasets	SVM	LASSO	SRC	SRC-LatLRR
Colon cancer	85.48	85.48	85.48	**90.32**
Prostate cancer	91.18	91.91	**94.85**	94.12
DLBCL	96.10	96.10	**97.40**	**97.40**

**Table 3:** Classification accuracies by different methods with gene selection for the three binary data sets.

Datasets	SVM	LASSO	SRC	SRC-LatLRR
Colon cancer (1000)	87.1	87.1	87.1	**91.94**
Prostate cancer (1500)	94.85	91.18	95.59	**96.32**
DLBCL (800)	97.40	93.51	97.40	**100**

**Table 4:** Descriptions of the four multiclass data sets used in DNA classification experiments.

Dataset	Class counts	Samples	Genes
Lung cancer	5	203	12600
Leukemia	3	72	11225
11_tumors	11	174	12533
9_tumors	9	60	5726

**Table 5:** Classification accuracies by different methods for the multiclass data sets.

Dataset	SVM	SRC	SRC-LatLRR
Lung cancer	**96.05**	95.07	95.07
Leukemia	96.60	95.83	**98.61**
11_tumors	94.68	**94.83**	**94.83**
9_tumors	65.10	**66.67**	**66.67**

**Table 6:** Classification accuracies by different methods with gene selection for the multiclass data sets.

Dataset	SVM	SRC	SRC-LatLRR
Lung cancer (2000)	**96.62**	95.07	95.57
Leukemia (3000)	96.90	95.83	**98.61**
11_tumors (1000)	**96.07**	95.40	95.40
9_tumors (2000)	**85.84**	71.67	80.00

## Abbreviations

SR: Sparse representation
SRC: Sparse representation classification
LRR: Low-rank representation
LatLRR: Latent low-rank representation
ALM: Augmented Lagrange multiplier
SVM: Support vector machines
LASSO: Least absolute shrinkage and selection operator
BW: Between-categories to within-category.

## References

[1] Cánovas A, Rincon G, Islas-Trejo A, Wickramasinghe S, Medrano JF (2010). SNP discovery in the bovine milk transcriptome using RNA-Seq technology. *Mammalian Genome*.

[2] Golub TR, Slonim DK, Tamayo P (1999). Molecular classification of cancer: class discovery and class prediction by gene expression monitoring. *Science*.

[3] Brunet JP, Tamayo P, Golub TR, Mesirov JP (2004). Metagenes and molecular pattern discovery using matrix factorization. *Proceedings of the National Academy of Sciences of the United States of America*.

[4] Gao Y, Church G (2005). Improving molecular cancer class discovery through sparse non-negative matrix factorization. *Bioinformatics*.

[5] Huang DS, Zheng CH (2006). Independent component analysis-based penalized discriminant method for tumor classification using gene expression data. *Bioinformatics*.

[6] Paul TK, Iba H (2009). Prediction of cancer Class with majority voting genetic programming classifier using gene expression data. *IEEE/ACM Transactions on Computational Biology and Bioinformatics*.

[7] Zheng CH, Zhang L, Ng TY, Shiu CK, Huang DS (2011). Metasample-based sparse representation for tumor classification. *IEEE/ACM Transactions on Computational Biology and Bioinformatics*.

[8] Furey TS, Cristianini N, Duffy N, Bednarski DW, Schummer M, Haussler D (2000). Support vector machine classification and validation of cancer tissue samples using microarray expression data. *Bioinformatics*.

[9] Lee YS, Krishnan A, Zhu Q, Troyanskaya OG (2013). Ontology-aware classification of tissue and cell-type signals in gene expression profiles across platforms and technologies. *Bioinformatics*.

[10] Tanic M, Andress E, Rodriguez-Pinilla SM (2013). MicroRNA-based molecular classification of non-BRCA1/2 hereditary breast tumours. *British Journal of Cancer*.

[11] Huang JH, Xie HL, Yan J, Lu HM, Xu QS, Liang YZ (2013). Rsing random forest to classify T-cell epitopes based on amino acid properties and molecular features. *Analytica Chimica Acta*.

[12] Nanni L, Brahnam S, Ghidoni S, Menegatti E, Barrier T (2013). Acomparison of methods for extracting information from the co-occurrence matrix for subcellular classification. *Expert Systems with Applications*.

[13] Lioyd GR, Almond LM, Stone N (2014). Utilising non-consensus pathology measurements to improve the diagnosis of oesophageal cancer using a raman spectroscopic probe. *The Analyst*.

[14] Braz G, da Rocha SV, Gattass M, Silva AC, de Paiva AC (2013). A mass classification using spatial diversity approaches in mammography images for false positive reduction. *Expert Systems with Applications*.

[15] Chen SS, Donoho DL, Saunders MA (2001). Atomic decomposition by basis pursuit. *SIAM Review*.

[16] Donoho DL (2006). Compressed sensing. *IEEE Transactions on Information Theory*.

[17] Wright J, Yang AY, Ganesh A, Sastry SS, Ma Y (2009). Robust face recognition via sparse representation. *IEEE Transactions on Pattern Analysis and Machine Intelligence*.

[18] Mairal J, Bach F, Ponce J, Sapiro G, Zisserman A Supervised dictionary learning.

[19] Liu GC, Lin ZC, Yan SC, Sun J, Yu Y, Ma Y (2013). Robust recovery of subspace structures by low-rank representation. *IEEE Transactions on Pattern Analysis and Machine Intelligence*.

[20] Liu G, Yan SC Latent low-rank representation for subspace segmentation and feature extraction.

[21] Liu GC, Lin ZC, Yu Y Robust subspace segmentation by low-rank representation.

[22] Liu GC, Xu H, Yan SC (2012). Exact subspace segmentation and outlier detection by low-rank representation. *JMLR: Workshop and Conference Proceedings*.

[23] Ma L, Wang CH, Xiao BH, Zhou W Sparse representation for face recognition based on discriminative low-rank dictionary learning.

[24] Candès EJ, Romberg J, Tao T (2006). Robust uncertainty principles: exact signal reconstruction from highly incomplete frequency information. *IEEE Transactions on Information Theory*.

[25] Candes EJ, Tao T (2006). Near-optimal signal recovery from random projections: universal encoding strategies?. *IEEE Transactions on Information Theory*.

[26] Tibshirani R (2011). Regression shrinkage and selection via the lasso: a retrospective. *Journal of the Royal Statistical Society B*.

[27] Kim SJ, Koh K, Lustig M, Boyd S, Gorinevsky D (2007). An interior-point method for large-scale *l*
_1_-regularized least squares. *IEEE Journal on Selected Topics in Signal Processing*.

[28] Candes EJ, Plan Y (2010). Matrix completion with noise. *Proceedings of the IEEE*.

[29] Candès EJ, Recht B (2009). Exact matrix completion via convex optimization. *Foundations of Computational Mathematics*.

[30] Keshavan RH, Montanari A, Oh S (2010). Matrix completion from noisy entries. *Journal of Machine Learning Research*.

[31] Fazel M (2002). *Matrix rank minimization with applications [Ph.D. thesis]*.

[32] Ni Y, Sun J, Yuan X, Yan S, Cheong LF Robust low-rank subspace segmentation with semidefinite guarantees.

[33] Lin Z, Chen M, Wu L, Ma Y (2009). The augmented lagrange multiplier method for exact recovery of corrupted low-rank matrices.

[34] Hang X, Wu FX (2009). Sparse representation for classification of tumors using gene expression data. *Journal of Biomedicine and Biotechnology*.

[35] Ghosh D, Chinnaiyan AM (2005). Classification and selection of biomarkers in genomic data using LASSO. *Journal of Biomedicine and Biotechnology*.

[36] Statnikov A, Aliferis CF, Tsamardinos I, Hardin D, Levy S (2005). A comprehensive evaluation of multicategory classification methods for microarray gene expression cancer diagnosis. *Bioinformatics*.

[37] Pochet N, de Smet F, Suykens JAK, de Moor BLR (2004). Systematic benchmarking of microarray data classification: assessing the role of non-linearity and dimensionality reduction. *Bioinformatics*.

[38] Alon U, Barka N, Notterman DA (1999). Broad patterns of gene expression revealed by clustering analysis of tumor and normal colon tissues probed by oligonucleotide arrays. *Proceedings of the National Academy of Sciences of the United States of America*.

[39] Singh D, Febbo PG, Ross K (2002). Gene expression correlates of clinical prostate cancer behavior. *Cancer Cell*.

[40] Shipp MA, Ross KN, Tamayo P (2002). Diffuse large B-cell lymphoma outcome prediction by gene-expression profiling and supervised machine learning. *Nature Medicine*.

[41] Bhattacharjee A, Richards WG, Staunton J (2001). Classification of human lung carcinomas by mRNA expression profiling reveals distinct adenocarcinoma subclasses. *Proceedings of the National Academy of Sciences of the United States of America*.

[42] Armstrong SA, Staunton JE, Silverman LB (2002). MLL translocations specify a distinct gene expression profile that distinguishes a unique leukemia. *Nature Genetics*.

[43] Su AI, Welsh JB, Sapinoso LM (2001). Molecular classification of human carcinomas by use of gene expression signatures. *Cancer Research*.

[44] Staunton JE, Slonim DK, Coller HA (2001). Chemosensitivity prediction by transcriptional profiling. *Proceedings of the National Academy of Sciences of the United States of America*.

